# Macrophage cholesterol efflux correlates with lipoprotein subclass distribution and risk of obstructive coronary artery disease in patients undergoing coronary angiography

**DOI:** 10.1186/1476-511X-8-14

**Published:** 2009-04-06

**Authors:** Patrick Linsel-Nitschke, Henning Jansen, Zouhair Aherrarhou, Stefanie Belz, Björn Mayer, Wolfgang Lieb, Fritz Huber, Werner Kremer, Hans-Robert Kalbitzer, Jeanette Erdmann, Heribert Schunkert

**Affiliations:** 1 Department of Medicine, University of Lübeck, Ratzeburger Allee 160, 23538 Lübeck, Germany; 2 Biophysics Institute, University of Regensburg, Josef Engert Strasse 9, 93053 Regensburg, Germany; 3 LipoFIT Analytics GmbH, Josef Engert Strasse 9, 93053 Regensburg, Germany

## Abstract

**Background:**

Studies in patients with low HDL have suggested that impaired cellular cholesterol efflux is a heritable phenotype increasing atherosclerosis risk. Less is known about the association of macrophage cholesterol efflux with lipid profiles and CAD risk in normolipidemic subjects. We have therefore measured macrophage cholesterol efflux in142 normolipidemic subjects undergoing coronary angiography.

**Methods:**

Monocytes isolated from blood samples of patients scheduled for cardiac catheterization were differentiated into macrophages over seven days. Isotopic cholesterol efflux to exogenously added apolipoprotein A-I and HDL2 was measured. Quantitative cholesterol efflux from macrophages was correlated with lipoprotein subclass distribution in plasma from the same individuals measured by NMR-spectroscopy of lipids and with the extent of coronary artery disease seen on coronary angiography.

**Results:**

Macrophage cholesterol efflux was positively correlated with particle concentration of smaller HDL and LDL particles but not with total plasma concentrations of HDL or LDL-cholesterol. We observed an inverse relationship between macrophage cholesterol efflux and the concntration of larger and triglyceride rich particles (VLDL, chylomicrons). Subjects with significant stenosis on coronary angiography had lower cholesterol efflux from macrophages compared to individuals without significant stenosis (adjusted p = 0.02).

**Conclusion:**

Macrophage cholesterol efflux is inversely correlated with lipoprotein particle size and risk of CAD.

## Introduction

The excessive uptake of lipoproteins by cells in the arterial wall gives rise to cholesterol-loaded cells with foamy cytoplasm due to the presence of cholesteryl ester droplets known as foam cells. These foam cells form the hallmark of atherosclerotic plaques. The efflux of cholesterol from foam cells is mediated by high-density lipoprotein (HDL) or its apolipoproteins and is widely believed to represent a crucial step in the prevention or reversal of atherosclerosis [1]. Macrophage cholesterol efflux has been proposed as the initial step in the reverse cholesterol transport (RCT), a prominent hypothesis to explain the anti-atherogenic mechanisms of HDL [2]. Although for many years the efllux of cholesterol was thought to occur primarily by passive aqueous diffusion, recently it has become clear that cholesterol efflux from macrophages is a highly regulated process that is mediated by specific molecules, including ATP-binding cassette transporters (ABC-transporters). Mutations in ABCA1 have been shown to cause Tangier-disease [3-6], a rare autosomal recessive disease which is characterized by extremely low plasma HDL-concentrations, the accumulation of foam cells in various tissues and moderately increased atherosclerosis suceptibilty. Importantly, patients with this condition show almost complete absence of cholesterol and phospholipid-efflux from macrophages [7].

In the mouse model, macrophage specific knockout of ABCA1 does not have a significant effect on HDL plasma levels but results in increased atherosclerosis [6,8,9]. While cholesterol efflux from macrophages might play a crucial role in foam cell development it has been demonstrated in the mouse model that ABCA1-mediated cholesterol efflux in the liver is primarily responsible in determining total plasma HDL[10,11].

In humans, a number of studies have examined cholesterol efflux from cultivated skin fibroblasts or monocyte derived macrophages isolated from the blood of patients with familial low HDL [12-16]. These studies suggest that defective cholesterol efflux from macrophages is a heritable cellular phenotype that cosegregates with low HDL.

In contrast to these findings, a recent study by Nakanishi and coworkers demonstrates that serum from patients with low HDL displays reduced cholesterol efflux capacity whereas no difference in cellular efflux capacity was found[17].

While the aforementioned studies have been conducted in subjects with the rare condition of familial low HDL, the correlation of macrophage cholesterol efflux with plasma lipids and coronary atherosclerosis in a normolipidemic population has not been examined so far. To address this problem, we have measured cholesterol efflux from monocyte-derived macrophages in 142 patients undergoing coronary angiography at our center and examined the relationship with plasma lipids, lipoprotein subclasses and coronary atherosclerosis.

## Methods

### Patient selection

We have randomly selected 142 patients undergoing scheduled diagnostic coronary angiography at our center between January 2005 and June 2007. Patients receiving lipid lowering medication were excluded from the study. All patients were fasting and blood was drawn by direct venipuncture at least one hour before cardiac catheterization was performed. Patients with acute coronary syndromes, inflammatory conditions or known neoplasms were excluded from the study. Plasma lipids were determined in the central laboratory of the Univerity of Lübeck Hospital. This study was conducted as part of the Lübeck Registry of Structural Heart Disease. All subjects gave informed consent and the study protocol was approved by the local ethics committee.

### Cholesterol efflux from monocyte derived macrophages

Peripheral blood mononuclear cells (PBMCs) were separated from whole blood by FICOLL-gradient, washed and resuspended in cell-culture medium. Cell culture medium used was DMEM supplemented with human serum from a single male donor, blood group ABRh- with the same charge being used throughout the study. 500.000 cells/well were plated out on a 12-well plate. After 1 hour all non-attached cells were removed by washing. Adherent cells were cultivated for 7 days with change of medium 24 hours and 4 days after isolation of monocytes. Differentiation of monocytes into macrophages was controlled in a subset of cases by scraping cells off the plate and performing flow-cytometric analysis of macrophage differentiation markers using the following monoclonals: CD16-FITC (Beckman-Coulter); CD64-FITC (Pharmingen), CD33-PE (Beckman); CD45PerCP (BD) and CD14-APC (BD).

On day seven, monocyte derived macrophages were incubated with ^3^H-labelled fetal-bovine-serum (FBS) Cholesterol (2 μCi/ml; Amersham) plus the LXR-agonist T0901317 (10 micromol/l, Sigma) for 24 hours followed by extensive washes. Cells were treated with the LXR-agonist T0901317 in order upregulate the capacity for cholesterol efflux and thus achieve a more stable experimental system[18].

Cells were then equilibrated with serum-free medium containing 0,2% Bovine Serum Albumin (BSA) for 30 min before either apo A-I (10 microg/ml), HDL2 (20 microg/ml) or a BSA-control (0,2%) were added into separate well. For each acceptor measurements were performed in triplicate. Lipid-free, recombinant apoA-I and HDL2 from the same donor were purchased from Biodesign (Saco, Maine), the same charge was used throughout the study. Cholesterol efflux from cells was stopped after 24 hours by collecting the medium and lysing the cells in 0,1 N sodium hydroxide containing 1% SDS. Cholesterol efflux was determined as % of total cellular radioactivity released into the medium. BSA mediated efflux was subtracted from HDL2 and apoA-I mediated efflux to calculate the efflux specific for each of these acceptors. Duplicate measurement of cholesterol efflux from the same individuals was carried out in 10% of cases. In these subjects, a second blood sample was obtained one day after cardiac catheterization and a separate cultivation of cells and cholesterol efflux assay was carried out.

### Plasma lipids

Blood samples from all patients were obtained during angiography. Plasma lipids (Total-, LDL-, HDL-cholesterol and triglycerides) were determined using standard fluorimetric methods at the central laboratory of the University of Lübeck hospital. Lipoprotein subclass distribution was assessed by NMR spectroscopy and carried out at LipoFit GmbH, Regensburg, Germany (patents: WO 2005/119285 A1, and DE 10 2004 026903 A1). Gradient-weighted NMR spectra of blood plasma were recorded on a Bruker 600 MHz spectrometer Avance II and revealed characteristic overall profiles of the lipoprotein signals. The spectral regions of the spectra ranging from 1.5 to 0.7 ppm were modelled into a set of 15 lipoprotein subclasses and classified according to additional file 1.

### Coronary angiography

All patients underwent coronary angiography and coronary angiograms were read by two experienced interventional cardiologists who were blinded for all other patient information. Coronary artery disease (CAD) with significant stenosis was defined as luminal narrowing of a major coronary artery greater than 50%. No CAD or non-obstructive CAD was defined by absence of lesions or by lesions without significant luminal narrowing of the coronary arteries (<50%)

## Results

### Characteristics of the patient population

142 patients (35 women, 107 men) undergoing coronary angiography at our center were included in this study. 90 individuals were affected from obstructive CAD with significant lesions resulting in luminal narrowing greater than 50% in one or more segments. The remaining 52 individuals showed either non-significant lesions or angiographic exclusion of CAD.

Baseline characteristics of the patient population are displayed in Additional file 1. Average plasma HDL-cholesterol, LDL-cholesterol and total cholesterol in the overall group were within normal limits. There was no statistically significant difference in plasma lipid or lipoprotein particle concentration between individuals affected by obstructive CAD and non-affected individuals with only age and gender showing significant differences in distribution between the groups.

### Correlation of macrophage cholesterol efflux with plasma HDL and lipoprotein subclass particle distribution

Overall, the quantitative macrophage cholesterol efflux to exogenously added apoA-I and HDL in the total population showed normal distribution and individual measurements were highly reproducible in separate experiments (data not shown).

Cholesterol efflux from cultivated monocyte derived-macrophages cultured ex-vivo for 7 days was subsequently compared to plasma lipid concentration and lipoprotein subclass distribution in blood samples obtained from the respective individual during angiography.

**Figure 1:**
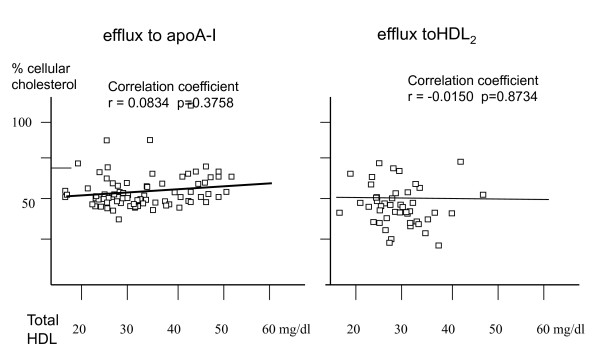
**Correlation of macrophage cholesterol efflux from patient derived macrophages onto exogenously added apoA-I (10 microgramm/ml; left chart) and HDL2 (20 microgramm/ml; right chart) with plasma HDL levels from a blood sample of respective individuals obtained at the time of coronary angiography**. Cholesterol efflux is expressed as % of total cellular radioactivity released into the medium after 24 hours incubation.

No significant correlation between macrophage cholesterol efflux and total plasma HDL-levels was observed for both apoA-I (Pearson correlation coefficient r = 0.08; p = 0.37) and HDL2 (r = -0.01; p = 0.87) dependent efflux (figure 1). Furthermore, there was no significant correlation of macrophage cholesterol efflux to total-cholesterol, LDL-cholesterol and triglycerides (data not shown).

**Figure 2:**
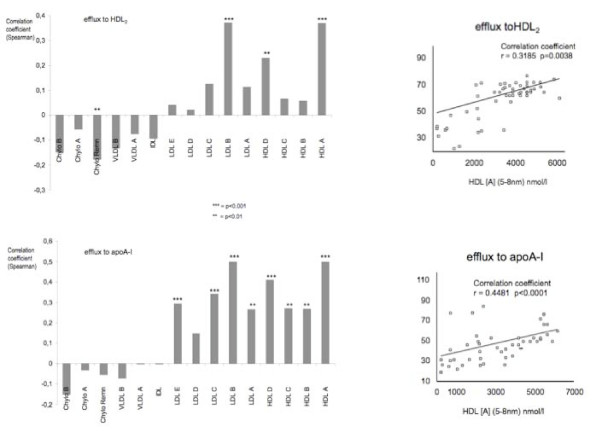
**Correlation of cholesterol efflux to HDL2 (top left) and apoA-I (bottom left) with concentration of lipoprotein particles in various subclasses as determined by NMR-spectroscopy of lipids**. For size definition of individual lipoprotein particles see Table 1. Correlation is expressed as Spearman's correlation coefficient (r) = y-axis with the level of significance indicated for each p < 0.01. Correlation of macrophage cholesterol efflux from patient derived macrophages onto exogenously added HDL2 (20 microgramm/ml; top right) and apoA-I (10 microgramm/ml; bottom right) with concentration of HDL [A] particles in nmol/l (HDL [A] = smallest subclass of HDL particles as defined by NMR with a size of 7 to 8.5 nm). Cholesterol efflux is expressed as % of total cellular radioactivity released into the medium after 24 hours incubation.

However, when macrophage cholesterol efflux was correlated with particle concentration in the whole range of lipoprotein subclasses, we observed a significant correlation between quantitative cholesterol efflux to apoA-I and HDL2 with particle concentration in several lipoprotein subclasses (Figure 2, left panels). In general, this correlation of macrophage cholesterol efflux to both apoA-I and HDL2 showed an inverse correlation to the size of lipoprotein particles (Figure 2, left panels). The correlation was positive for the smaller and more cholesterol rich lipoprotein subclasses (HDL [A] through HDL [D] and LDL [A] through LDL [E]. In contrast, the correlation proved negative for the comparably larger and triglyceride rich lipoprotein subclasses (Chylomicrons, VLDL and IDL).

The strongest positive correlation between macrophage cholesterol efflux to both apoA-I and HDL2 as acceptors concentration was observed for the smallest group of HDL-particles (HDL [A]: r = 0.50; p = 0.0001 for efflux to apoA-I; r = 0.37; p = 0.0001 for efflux to HDL2). The correlation between individual values for cholesterol efflux to apoA-I and HDL2 with particle concentration of HDL [A] in plasma from the same individuals is dispayed in figure 2 (right panels).

### Association of macrophage cholesterol efflux with obstructive CAD

Since impaired cholesterol efflux from macrophages has been suggested to contribute to atherosclerosis development, we next examined whether a quantitative difference in macrophage cholesterol efflux between subjects affected from obstructive CAD and those subjects with no CAD or only mild disease was detectable. For cholesterol efflux to HDL2 the average percentage of cholesterol released into the medium in patients with obstructive CAD (n = 90) was 46.1% (95% CI: 5,0%, StdDev: 18.5%) compared to 50.9% (95%CI: 2.8, StdDev: 13.7%) in patients without CAD or with non-obstructive lesions (n = 52).

**Figure 3:**
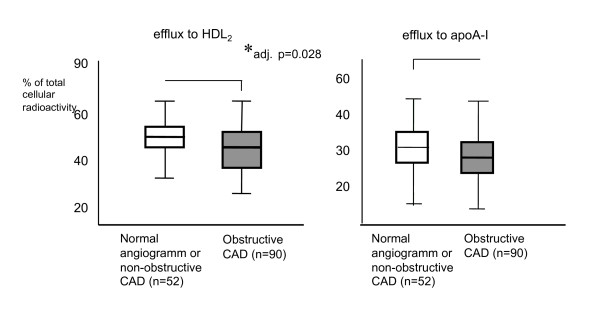
**Comparison of macrophage cholesterol efflux from patient derived macrophages onto exogenously added HDL2 (20 microgramm/ml; left chart) and apoA-I (10 microgramm/ml; right chart) according to CAD status**. Obstructive CAD was defined as presence of lesions resulting in luminal narrowing >50% in one or more coronary artery. Displayed are average cholesterol efflux, 95% confidence interval (boxes) and standard deviation (error bar). *0 = 0.025 for cholesterol efflux to HDL2 in patients with obstructive CAD versus controls in a univariate regression analysis adjusting for age and sex.

For cholesterol efflux to apoA-I as acceptor the average percentage of cellular cholesterol released was 29.8% in patients with obstructive CAD (95%CI: 4.2, StdDev: 15.6) compared to 31.3% (95%CI: 3.0, StdDev: 14.35) in the no CAD/non obstructive group (Figure 3).

Since male sex and age were risk factors significantly associated with risk of obstructive CAD in the baseline characteristics of our study population these risk factors were included into a regression analysis. After adjusting for sex and age we found a significant inverse correlation between HDL2-mediated macrophage cholesterol efflux and obstructive CAD on coronary angiogram (p = 0.02, Figure 3). While a similar inverse correlation was observed for efflux to apoA-I as well, it did not reach statistical significance.

## Discussion

Previous studies suggest that cholesterol efflux from human cells grown in primary culture presents a heritable cellular phenotype with functional correlation to lipid metabolism and potentially the risk of coronary atherosclerosis. These studies were mainly undertaken in small numbers of individuals suffering from familial HDL deficiency syndromes. In contrast, our study was conducted in a group of normolipidemic individuals undergoing scheduled coronary angiography. In this group we have studied the correlation of macrophage cholesterol efflux with plasma lipids, lipoprotein subclass distribution and angiographic extent of CAD. Our study hypothesis is based on the results of previous studies suggesting that genetically determined differences in cholesterol efflux and other cellular functions are preserved in cells cultivated ex-vivo. Accordingly, if cells isolated from subjects will have a stable functional phenoype over 7 days, it should be informative to compare lipid profiles and angiography results from these individuals obtained at baseline with cholesterol-efflux results measured after 7 days of culture.

In contrast to a previous study conducted in patients with familial low HDL [15,16], we did not observe a significant correlation between cholesterol efflux from macrophages and plasma HDL concentration. A possible explanation for this finding could be that macrophage cholesterol efflux is quantitatively less important to maintain plasma HDL levels in a normolipidemic population compared to patients with extremely low HDL. Since animal studies have also demonstrated that macrophage cholesterol efflux does not have a substantial impact on plasma HDL[6,8,9], our study supports the hypothesis that cholesterol efflux from other tissues such as for example liver or muscle is more important to determine quantitative HDL levels.

Because more subtle effects of macrophage cholesterol efflux on the reverse cholesterol transport might not be detectable by measuring total HDL levels, we used NMR spectroscopy of plasma lipids to determine the lipoprotein subclass distribution in plasma samples of all subjects in which macrophage cholesterol efflux had been measured. In large scale clinical studies involving human subjects NMR spectroscopy of lipids has been demonstrated as a reliable method to characterize lipoprotein subclass distribution in plasma[15]. Interestingly, we observed an inverse correlation between macrophage cholesterol efflux and lipoprotein particle size on NMR spectroscopy. While the concentration of large triglyceride-rich lipoprotein particles such as chylomicron-remnants or VLDL was negatively correlated with macrophage cholesterol efflux we found a strong positive correlation efflux with smaller, more cholesterol rich particles in the HDL and LDL fraction. This finding seems functionally plausible, since HDL-particles in particular and to a lesser extent also LDL-particles have been shown to promote cellular cholesterol efflux in cell-based assays and in vivo. The positive correlation was particularly strong for the smallest subclass of HDL particles measured by NMR, which might resemble the lipid-poor pre-β HDL particles. These pre-β HDL particles are widely believed to act as the preferential substrate for ABCA1-mediated cholesterol efflux, which has been proposed as the initiating step for the reverse cholesterol transport. The inverse relationship of macrophage cholesterol efflux with large triglyceride-rich lipoproteins (TRLs) does also fit with physiological models in which plasma HDL, HDL-turnover and lipolytic-activity are negatively correlated with TRL-concentration[19].

Measuring the concentration of particles in lipoprotein subclasses might therefore better represent the dynamic flux in the reverse cholesterol pathway than the more static determination of total HDL, LDL or triglyceride levels.

Macrophage cholesterol efflux is believed to be anti-atherogenic by preventing or reversing the development of foam cells in the arterial wall. Since the correlation between angiographic CAD and macrophage cholesterol efflux has not been examined so far, we next investigated whether a quantitative difference in cholesterol efflux could be detected between the group of patients with obstructive CAD and the group without CAD or non-obstructive lesions.

Indeed, after adjusting for sex and age we found a significant inverse correlation between HDL2-mediated macrophage cholesterol efflux and obstructive CAD. For apoA-I mediated efflux a similar trend was observable that did not reach statistical significance.

Macrophages were preconditioned equally during the preparation, so that it seems unlikely for these cells to be influenced by environmental conditions or by the pathological stage they were exposed to in CHD. The data therefore reflect more fundamental background of these cells such as genetic factor that might cause the pathological status. Generally speaking, the apoA-I-dependent release should closely be associated with function of ABCA1, and the HDL2-dependent release should be associated with ABCG1-function or other factors that regulate the exchange-based non-specific release. In such a view, positive correlation of the apoA-I-mediated release to HDL3 concentration may indicate that HDL3 levels reflect basic capability of cells to generate HDL, such as activity of ABCA1. The similar correlation of the HDL2-mediated release may imply that expression of ABCG1 could be parallel to ABCA1 expression. From the pathophyiological point of view, the ABCG1-dependent cholesterol release might be more important than the ABCA1-dependent release.

Our study has several limitations that need to be taken into account when interpreting these results. Methods to cultivate monocyte-derived macrophages and determine cellular cholesterol efflux are at present extremely labour- and cost-intensive and thus preclude larger case numbers. We have included 142 subjects and our study is therefore, to our knowledge, the largest cohort in which cellular cholesterol efflux from patient derived cells was assessed. While it is noteworthy that a difference in means between cases and controls was detectable in our study, it will be essential to replicate these findings in larger cohorts in the future in order to further substantiate the role of macrophage cholesterol efflux as a CAD risk factor.

## Conclusion

The finding that macrophage cholesterol efflux from patient-derived cells measured ex-vivo in a serum-free system correlates to lipoprotein particle distribution in plasma and potentially to CAD risk indicates that this cellular mechanism is to a large degree genetically determined. Identification of novel genes regulating macrophage cholesterol efflux will be an important aim of future research in which functional studies using patient derived cells can be a valuable tool.

## Competing interests

The authors declare that they have no competing interests.

## Authors' contributions

HJ, ZA, SB and PLN carried out cell culture works and cholesterol efflux assays. HJ, BM, WL and HS recruited patients for this study. HJ, WL and PLN carried out data analysis. HJ, JE, HS and PLN designed the study and wrote the manuscript. FH, WK and HRK. performed NMR-spectroscopy of lipids.

## Supplementary Material

Additional File 1 **Baseline characteristics of the study population**. Demographics and details of lipid measurements for the study population. Click here for file

## References

[B1] Linsel-Nitschke P, Tall AR (2005). HDL as a target in the treatment of atherosclerotic cardiovascular disease. Nat Rev Drug Discov.

[B2] Glomset JA (1980). High-density lipoproteins in human health and disease. Adv Intern Med.

[B3] Bodzioch M, Orso E, Klucken J, Langmann T, Bottcher A, Diederich W, Drobnik W, Barlage S, Buchler C, Porsch-Ozcurumez M, Kaminski WE, Hahmann HW, Oette K, Rothe G, Aslanidis C, Lackner KJ, Schmitz G (1999). The gene encoding ATP-binding cassette transporter 1 is mutated in Tangier disease. Nat Genet.

[B4] Brooks-Wilson A, Marcil M, Clee SM, Zhang LH, Roomp K, van Dam M, Yu L, Brewer C, Collins JA, Molhuizen HO, Loubser O, Ouelette BF, Fichter K, Ashbourne-Excoffon KJ, Sensen CW, Scherer S, Mott S, Denis M, Martindale D, Frohlich J, Morgan K, Koop B, Pimstone S, Kastelein JJ, Hayden MR (1999). Mutations in ABC1 in Tangier disease and familial high-density lipoprotein deficiency. Nat Genet.

[B5] Rust S, Rosier M, Funke H, Real J, Amoura Z, Piette JC, Deleuze JF, Brewer HB, Duverger N, Denefle P, Assmann G (1999). Tangier disease is caused by mutations in the gene encoding ATP-binding cassette transporter 1. Nat Genet.

[B6] Aiello RJ, Brees D, Bourassa PA, Royer L, Lindsey S, Coskran T, Haghpassand M, Francone OL (2002). Increased atherosclerosis in hyperlipidemic mice with inactivation of ABCA1 in macrophages. Arterioscler Thromb Vasc Biol.

[B7] Francis GA, Knopp RH, Oram JF (1995). Defective removal of cellular cholesterol and phospholipids by apolipoprotein A-I in Tangier Disease. J Clin Invest.

[B8] Haghpassand M, Bourassa PA, Francone OL, Aiello RJ (2001). Monocyte/macrophage expression of ABCA1 has minimal contribution to plasma HDL levels. J Clin Invest.

[B9] Downes M, Verdecia MA, Roecker AJ, Hughes R, Hogenesch JB, Kast-Woelbern HR, Bowman ME, Ferrer JL, Anisfeld AM, Edwards PA, Rosenfeld JM, Alvarez JG, Noel JP, Nicolaou KC, Evans RM (2003). A chemical, genetic, and structural analysis of the nuclear bile acid receptor FXR. Mol Cell.

[B10] Ragozin S, Niemeier A, Laatsch A, Loeffler B, Merkel M, Beisiegel U, Heeren J (2005). Knockdown of hepatic ABCA1 by RNA interference decreases plasma HDL cholesterol levels and influences postprandial lipemia in mice. Atheroscler Thromb Vasc Biol.

[B11] Timmins JM, Lee JY, Boudyguina E, Kluckmann KD, Brunham LR, Mulya A, Gebre AK, Coutinho JM, Colvin PL, Smith TL, Hayden MR, Maeda N, Parks JS (2005). Targeted inactivation of hepatic Abca1 causes profound hypoalphalipoproteinemia and kidney hypercatabolism of apoA-I. J Clin Invest.

[B12] Marcil M, Bissonnette R, Vincent J, Krimbou L, Genest J (2003). Cellular phospholipid and cholesterol efflux in high-density lipoprotein deficiency. Circulation.

[B13] van Dam MJ, de Groot E, Clee SM, Hovingh GK, Roelants R, Brooks-Wilson A, Zwinderman AH, Smit AJ, Smelt AH, Groen AK, Hayden MR, Kastelein JJ (2002). Association between increased arterial-wall thickness and impairment in ABCA1-driven cholesterol efflux: an observational study. Lancet.

[B14] Hovingh GK, Van Wijland MJ, Brownlie A, Bisoendial RJ, Hayden MR, Kastelein JJ, Groen AK (2003). The role of the ABCA1 transporter and cholesterol efflux in familial hypoalphalipoproteinemia. J Lipid Res.

[B15] Soro-Paavonen A, Naukkarinen J, Lee-Rueckert M, Watanabe H, Rantala E, Soderlund S, Hiukka A, Kovanan PT, Jauhiainen M, Peltonen L, Taskinen MR (2007). Common ABCA1 variants, HDL levels, and cellular cholesterol efflux in subjects with familial low HDL. Journal of Lipid Research.

[B16] Kiss RS, Kavaslar N, Okuhira K, Freeman MW, Walter S, Milne RW, McPherson R, Marcel YL (2007). Genetic etiology of isolated low HDL syndrome: incidence and heterogeneity of efflux defects. Arterioscler Thromb Vasc Biol.

[B17] Nakanishi S, Vikstedt R, Söderlund S, Lee-Rueckert M, Hiukka A, Ehnholm C, Muilu M, Metso J, Naukkarinen J, Palotie L, Kovanen PT, Jauhiainen M, Taskinen MR (2009). Serum, but not monocyte macrophage foam cells from low HDL-C subjects displays reduced cholesterol effluc capacity. Journal of Lipid Research.

[B18] Zanotti I, Poti F, Pedrelli M, Favari E, Moleri E, Franceschini G, Calabresi L, Bernini F (2008). The LXR agonist T0901317 promotes the reverse cholesterol transport from macrophages by increasing plasma efflux potential. J Lipid Res.

[B19] Patsch J (1998). Influence of lipolysis on chylomicron clearance and HDL cholesterol levels. Eur Heart J.

