# Supplementary material for: Macrophage cholesterol efflux correlates with lipoprotein subclass distribution and risk of obstructive coronary artery disease in patients undergoing coronary angiography
Source: Lipids Health Dis. 2009 Apr 6;8:14. doi: 10.1186/1476-511X-8-14 (PMC2674428; doi:10.1186/1476-511X-8-14)
Supplement: Additional File 1 — Baseline characteristics of the study population. Demographics and details of lipid measurements for the study population. [file 1476-511X-8-14-S1.pdf]

Table 1

Demographics/Risk factors	All		Obstructive CAD		No CAD/ non significant lesions		p-value
	mean	StD	mean	StD	mean	StD	
n	142		90		52		
Age (years)	66	11	68	9	62	11	0,001
male sex (%)	75		83	na	62	na	0,003
BMI	28,5	5	28,8	4,9	27,9	5	ns
Hypertension (%)	24		24		14		ns
Diabetes (%)	8		21		9		ns
Smoking (%)	6		24		8		ns
Total Cholesterol (mmol/l)	5,0	1,3	4,9	1,2	5,4	1,4	ns
LDL-Cholesterol (mmol/l)	2,9	0,9	2,9	0,9	3,1	0,9	ns
HDL-Cholesterol (mmol/l)	1,4	0,4	1,4	0,3	1,4	0,4	ns
Triglycerides (mmol/l)	1,7	0,9	1,6	0,9	1,9	1	ns
**Particle concentration (nmol/l)**	mean	95%CI	mean	95%CI	mean	95%CI	
Chylo. [B] (>150nm)	0,86	0,1	0,93	0,12	0,74	0,1	ns
Chylo. [A] (100-150nm)	0,05	0,0	0,04	0,02	0,06	0,0	ns
Chylo. Rem (80-100nm)	1,82	0,4	2,04	0,44	1,42	0,3	ns
VLDL [B] (60-80nm)	11,72	1,8	13,21	1,95	9,09	1,4	ns
VLDL [A] (40-60nm)	0,93	0,6	1,27	0,76	0,33	0,2	ns
IDL (30-40nm)	49,14	18,8	56,44	21,1	36,18	13,9	ns
LDL [E] (25-30nm)	101,45	10,6	103,27	11	98,2	9,8	ns
LDL [D] (22-25nm)	96,20	15,2	102,64	16,9	84,75	11,2	ns
LDL [C] (22-25nm)	157,49	21,6	161,72	23,2	149,98	18,8	ns
LDL [B] (19-21nm)	76,80	15,6	72,18	14	85	18,2	ns
LDL [A] (16-19nm)	50,30	12,2	46,17	12,53	57,64	11,7	ns
HDL [D] (13-16nm)	378,68	66,3	355,12	63,8	420,51	70,9	ns
HDL [C] (10-13nm)	964,55	149,8	974,3	155,5	947,24	140,6	ns
HDL [B] (8.5nm-10nm)	1262,69	202,2	1352,65	218,3	1102,97	170,8	ns
HDL [A] (7nm-8.5nm)	470,50	113,3	455,66	113,4	496,85	114,3	ns
